# From Evidence-Based Medicine to Evidence-Based Health: the Example of Asthma

**Published:** 2011-10-15

**Authors:** David Moskowitz, Thomas Bodenheimer

**Affiliations:** Division of General Internal Medicine, University of California San Francisco. Dr Moskowitz is also affiliated with San Francisco General Hospital, San Francisco, California; Center for Excellence in Primary Care, Department of Family and Community Medicine, University of California San Francisco, San Francisco, California

The reigning paradigm underlying the work of physicians is evidence-based medicine (EBM). Since the early 1990s, evidence-based clinical practice guidelines addressing the management of asthma have been widely disseminated. In a 2009 survey of US primary care physicians, 78% reported using asthma guidelines (1). Despite an array of clinical interventions rooted in EBM, asthma attacks, emergency department visits, and hospitalizations remain high for both adults and children (2). Furthermore, wide racial disparities in asthma-related health care use and mortality rates persist (2). These facts suggest that the advent of EBM has not solved the problem of preventing and managing asthma.

Even if evidence-based guidelines were completely implemented, EBM has 2 fundamental shortcomings. First, for asthma — as for any chronic illness — patients themselves make the day-to-day decisions. This fact has been incorporated into the Chronic Care Model through self-management support; it provides patients with the knowledge, skills, and confidence needed to better self-manage. Second, the physical and social environment in which patients live shapes both the risk factors they are exposed to (primary prevention) and their ability to effectively self-manage (secondary prevention). Our hypothesis is that, to be effective, EBM needs to be supplemented by self-management support that extends beyond the clinic and into the community and by interventions that change elements of the environment in which patients live (ie, community health interventions). The total of these 3 components can be called evidence-based health (EBH): EBM + self-management support + community health interventions = EBH. EBH acknowledges that evidence-based health care is insufficient to achieve desired health outcomes.

Asthma is a disease caused by the interaction of genetics and environment, and environmental factors may be largely responsible for the worsening asthma burden. Furthermore, low adherence to inhaled corticosteroids is pervasive, and health literacy, which is lower among people living in low-income communities, affects self-management. The combination of poverty, urban residence, and minority status underpins the contributions of the physical and social environment to the burden of asthma (3). Asthma, then, is a case study through which we can explore the paradigm shift from EBM to EBH.

Self-management support in the clinical setting (information, skills training, confidence building, and follow-up) is effective in reducing asthma symptoms and asthma-related emergency department visits (4). Self-management support interventions in the community can also be effective. For example, recently discharged African American patients who were assisted by asthma coaches had lower rates of rehospitalization than did similar patients who did not have coaches (5). Nurse-provided asthma education and referral to community resources, plus in-home environmental assessments, asthma education, and social support by community health workers, significantly increased symptom-free days among people with asthma compared with those who used only nurse-provided education (6). A Cochrane review of 32 studies evaluating community asthma education programs targeting children found improvement in several measures of asthma severity and functional status (7).

Among chronic conditions, asthma is unique in its relation to the physical environment. Specific triggers common to households (eg, mites, dust, mold) can provoke asthma exacerbations. Accordingly, many studies have investigated altering specific elements of the physical environment to reduce exposures. Although limited interventions targeting home environmental exposures in inner-city children with asthma have not reduced asthma severity, interventions reducing multiple triggers have improved asthma symptoms (8,9).

The Seattle-King County Healthy Homes Project employed community health workers to provide both education and environmental trigger reduction, comparing a high-intensity (7 home visits and a full set of environmental control resources) and low-intensity (1 home visit and limited resources) intervention. The high-intensity intervention was associated with significantly greater improvement in a pediatric asthma quality-of-life score, asthma-related urgent health service use, and allergen burden compared with the low-intensity intervention (10).

Substantial evidence supports that asthma outcomes can be improved by providing self-management support in the medical practice and extending such support to the community and particularly to the home. Educational programs and physical methods of environmental allergen reduction are both effective, as is the combination of education and allergen control. Evidence regarding allergen reduction is mixed, but reductions of global allergen burden in the home appear to be effective. Allergen reduction can be seen as both primary and secondary prevention, helping people who have asthma and averting asthma in the susceptible but asymptomatic population.

The studies presented here indicate that self-management support in the community and changes to the environment in which patients live are needed to improve the unsatisfactory results that are produced by EBM alone to improve asthma prevention and management. Improved asthma control and prevention require implementing a broad definition of health (ie, EBH) that extends beyond the clinic doors. This concept is not new but, in an era of increasing rates of chronic disease and widening disparities, must be revisited and translated into action. Clinical teams caring for patients with asthma can take some initial steps to implement EBH. First, teams must familiarize themselves with appropriate community resources, in particular related to avoiding triggers in housing, and engage these resources for their patients. Second, clinical teams and practice groups must advocate for better housing and cleaner air: such policy changes directly affect the health of their patients. Third, and perhaps most important, practices and providers must initiate discussions with insurers regarding reimbursement for these community health interventions, which reduce costs through reduced emergency department visits and hospital admissions, providing a business case for insurers to invest in evidence-based health (4,8,10). Implementing evidence-based health is not simple, but it is essential to reduce the burden of asthma and other chronic diseases and to help control the associated costs to society.
